# The use of epinephrine in out-of-hospital cardiac arrest: A retrospective study of the effects of administration timing and cumulative doses on outcome in a physician-staffed emergency medical service system

**DOI:** 10.1186/s12873-025-01351-4

**Published:** 2025-09-22

**Authors:** Tobias Gruebl, Janine Jungblut, Dennis Rupp, Chung Shing Rex Ha, Birgit Ploeger, Dana Maresa Haag, Davut Deniz Uzun, Christoph Walter Jaenig, Martin Christian Sassen, Bernhard Schieffer, Lars Timmermann, Susanne Betz

**Affiliations:** 1 Department of Anesthesiology and Intensive Care Medicine, Faculty of Medicine, Philipps University of Marburg, Baldingerstraße, Marburg, 35043 Germany; 2 Department of Anaesthesiology, Intensive Care Medicine, Emergency Medicine and Pain Therapy, Bundeswehr Central Hospital, Koblenz, Germany; 3 Department of Geriatrics, Varisano Main-Taunus Hospitals, Hofheim a.T, Germany; 4 Centre for Emergency Medicine, Gießen and Marburg University Hospital, Marburg, Germany; 5 German Red Cross, Emergency Medical Service Mittelhessen, Marburg, Germany; 6 Institute of Medical Biometry, Epidemiology and Informatics, University of Mainz Medical Centre, Mainz, Germany; 7 Medical Faculty Heidelberg, Department of Anaesthesiology, Heidelberg University, Heidelberg, Germany; 8 Hazard Prevention Services, District of Marburg-Biedenkopf, Marburg, Germany; 9 Department of Cardiology, Angiology, and Medical Intensive Care, Gießen and Marburg University Hospital, Marburg, Germany; 10 Department of Neurology, Gießen and Marburg University Hospital, Marburg, Germany

**Keywords:** Out-of-hospital cardiac arrest, Advanced life support, Epinephrine, Adrenaline, Outcome

## Abstract

**Background:**

Epinephrine administration during cardiopulmonary resuscitation (CPR) has been a long-standing recommendation, but the evidence is controversial. This study investigated effects of epinephrine administration in a physician-staffed emergency medical service (EMS) system and for the first time addressed the quality of chest compressions.

**Methods:**

Complete datasets of adult patients who suffered out-of-hospital cardiac arrest (OHCA) and received CPR were retrospectively analysed. Factors (time of collapse, bystander CPR, EMS arrival, initial cardiac rhythm, suspected cause of OHCA, and for the first time also quality of chest compressions) that may influence outcome (return of spontaneous circulation [ROSC], survival to discharge, neurological status) and epinephrine administration (time of first administration, total dose, route of administration) were analysed after adjustment.

**Results:**

A total of 1141 patients were identified; 1090 patients were included. Patient data (age, gender, pre-existing conditions, initial electrocardiographic rhythm, suspected cause) were comparable to those reported in international studies. Mean chest compression depth was 5.5 cm (SD: 0.8 cm). Median compression rate was 115/min (SD: 12/min). The first epinephrine dose was administered after a mean period of 6:43 min after EMS arrival (SD: 9:30 min) and 18:23 min after collapse (SD: 11:13 min). Earlier epinephrine administration was associated with increased rates of ROSC and survival to discharge. Patients who achieved ROSC and survived to discharge received less than 6 mg of epinephrine. Early administration was associated with improved outcomes, especially in patients with asystole. Neurological outcomes, however, deteriorated with increasing epinephrine doses.

**Conclusions:**

This study supports the benefit of early administration of limited doses of epinephrine in OHCA patients. Higher epinephrine doses may be associated with poorer outcomes. Further randomised controlled studies that investigate the administration of medications within fifteen minutes after collapse and also address the quality of basic life support measures are required to assess the actual benefits of epinephrine during CPR.

**Supplementary Information:**

The online version contains supplementary material available at 10.1186/s12873-025-01351-4.

## Graphical Abstract

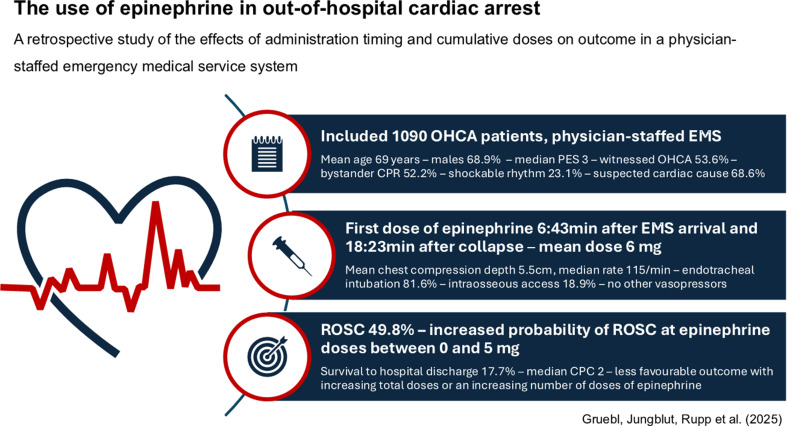

## Introduction

Acute cardiac arrest is one of the leading causes of death in industrial nations [1–3]. More than 75% of patients have a non-shockable rhythm on the initial electrocardiogram (ECG), and this proportion has been increasing in recent years [4–7]. Current guidelines on cardiopulmonary resuscitation (CPR) continue to recommend the immediate administration of epinephrine as a vasopressor agent, especially in patients with a non-shockable electrocardiographic rhythm [8]. Although there is some evidence supporting the effectiveness of epinephrine in patients with cardiac arrest, the actual impact of epinephrine on survival remains controversial [9]. The probability of survival from out-of-hospital cardiac arrest is generally low, approximately 10% in Europe. This is attributed to the fact that the causes of cardiac arrest are often severe and cardiac arrest is most commonly seen in elderly patients with serious pre-existing conditions [10–14]. The overall performance of the emergency medical services (EMS) team attempting to achieve return of spontaneous circulation (ROSC) as rapidly as possible plays a key role in the survival of patients with potentially reversible causes of cardiac arrest. If medications that provide circulatory support (e.g. epinephrine) are used in these patients, it is reasonable to administer them early and at a sufficient dose. Previous basic interventions such as chest compressions, oxygenation, ventilation, and vascular access are, however, required [8, 15]. Especially if cardiac arrest occurs in the out-of-hospital setting, the time of epinephrine administration and also the probability of survival are influenced by a variety of factors. Favourable factors are witnessed collapse and early call for medical assistance, bystander CPR, early arrival of EMS personnel, access to the patient, and the availability of material for the administration of medications [16–19]. Currently there are some robust data that allow the optimal time of epinephrine administration and/or the optimal dose of epinephrine during CPR to be determined depending on the route of administration.

The aim of this study was to obtain further data on the effects of epinephrine administration in patients with out-of-hospital cardiac arrest (OHCA) at different early time points. A further aim was to investigate a possible correlation between the probability of survival (including neurological status after survival) and the total dose of epinephrine. Unlike other studies, this analysis included both routine physician presence in the prehospital setting and, for the first time, an evaluation of chest compression quality during resuscitation. Based on the latest scientific findings, the quality of basic life support plays a decisive role, for example in distributing medications in the body [15]. In addition, the presence of a physician at the scene may lead to improved survival in these high-risk patients because physicians are highly trained and can administer medications in a more targeted way or can treat the causes of cardiac arrest earlier [20]. The treatment by an emergency physician as a part of the EMS is a special point of this study, especially in comparison with other international studies.

## Methods

Following approval from the responsible ethics committee, this study was conducted in a representative EMS area, which covers 1263 km^2^ with a population density of 199/km^2^ and which is served by a physician-staffed EMS. All patients who were treated for OHCA in the prehospital setting were identified using specific patient referral codes, which are always used in the study area to notify hospitals of incoming emergency patients or to complete digital patient records regardless of whether or not patients are transferred to a hospital. There are four different codes for cardiac arrest patients: (1) resuscitation (continuous/intermittent), (2) resuscitation (sustained ROSC), (3) resuscitation for trauma (continuous/intermittent), and (4) resuscitation for hypothermia. The EMS team at the scene selects one of these codes and patients are then usually transferred to a local cardiac arrest centre (CAC) [21]. Patient data were anonymised. Patients under the age of 18 years and patients with incomplete datasets were excluded. Data were extracted from EMS reports following the Utstein style [22]. Pre-existing medical conditions were assessed using the pre-emergency status (PES). The PES is a score for assessing the severity of pre-existing conditions of emergency patients, especially in the EMS setting, and is similar to the criteria of the American Society of Anaesthesiology (ASA) for the preoperative assessment of patients [23, 24]. A PES score ≥ 3 is defined as a severely impaired health status. Data on the time of first vasopressor administration and the cumulative total dose of epinephrine given during cardiac arrest were collected from EMS reports. Data on the rate and depth of chest compressions were obtained from a resuscitation feedback system (CPR feedback, Corpuls, Kaufering, Germany). According to the local protocol, the intraosseous (IO) route is used in the resuscitation setting if two attempts at intravenous (IV) access have failed or a venous puncture site has not been found within two minutes. In the EMS area that was analysed in this study, all EMS vehicles that are staffed with an emergency physician are operated by a single organisation. All vehicles were therefore equipped with essentially the same medical technical equipment and EMS personnel had essentially the same qualifications. The outcome of treatment in OHCA patients was documented. The neurological status of patients who were hospitalised and survived the cardiac arrest event was assessed using the five-point Cerebral Performance Category (CPC) scale. CPC scores of 1 and 2 are defined as a favourable neurological outcome [25]. The initial cardiac rhythm was used in the analysis of different subgroups. In accordance with European guideline recommendations, a combination of epinephrine and amiodarone was administered to patients with shockable rhythms after three defibrillation attempts. By contrast, patients with non-shockable electrocardiographic rhythms underwent this treatment as soon as possible.

Depending on the levels of measurement and the distribution of variables, means or medians and standard deviations or mean deviations from the median were used as measures of central tendency, minimum and maximum values as extreme values, and quartiles and interquartile ranges as descriptive measures. Based on Bayesian hypothesis testing, results were tested for statistical significance using the Bayes factor. In addition, regression analysis was performed using Bayesian logistic regression and Bayesian additive regression trees (BART) [26]. In the multivariate analysis, the effects of epinephrine administration were adjusted for age, sex, PES, ECG, suspected causes of cardiac arrest, and witnessed collapse. A Bayes factor > 5 was interpreted as a significant difference and > 100 as a highly significant difference.

## Results

From 1 January 2014 to 30 December 2018, a total of 1141 patients were treated for OHCA. Following the application of exclusion criteria, 1090 OHCA patients were included in the final analysis. Patients had a mean age of 69 years (SD: 14 years), 751 (68.9%) were male. The median PES score was 3. OHCA was witnessed in 53.6% of cases (*n* = 584), resuscitation was performed by bystanders in 52.2% (*n* = 569). Patients presented with shockable ECG rhythm (ventricular fibrillation, VF; pulseless ventricular tachycardia, pVT) in 23.1% (*n* = 252), with pulseless electrical activity (PEA) in 23.4% (*n* = 255), and asystole in 53.5% (*n* = 583) on first ECG analysis. A cardiac cause of OHCA was suspected in 68.6% (*n* = 748) of cases, hypoxia in 12.8% (*n* = 140), and trauma in 3.4% (*n* = 37). The reason for cardiac arrest was not identifiable in 15.1% (*n* = 165). Advanced airway management was performed in 87.7% (*n* = 956), endotracheal intubation in 81.6% (*n* = 886). Table 1 provides details on the quality of chest compressions. The feedback system showed that, in approximately 40% of cases, compression rates and depths were within the target ranges specified in guidelines. Compression depths exceeded 6 cm in about 30%, and chest compressions were performed at a rate of more than 120/minute in approximately 40%. There were no significant differences in compliance with guideline recommendations for compression depth (5–6 cm) and rate (100–12/minute) between patients with no ROSC and patients who survived to hospital discharge (depth: 41.89% vs. 37.70%, BF = 0.24; rate: 49.88% vs. 44.59%, BF = 0.27). OHCA patients received a median epinephrine dose of 5 mg (IQR 1–9 mg). The normality test on epinephrine doses rejected an assumption of normality (*p* < 2,2 × 10^− 16^). The first dose was administered to patients after an average of 6:43 min after EMS arrival (SD: 9:30 min) and 18:23 min after collapse (SD: 11:13 min). IO access was used in 18.9% (*n* = 206). In these cases, the first dose of epinephrine was administered early (7:14 min, SD: 5:49 min, 17:58 min, SD: 7:06 min, BF < 5 for both groups). When the IO route was used, the quality of chest compressions (depth and rate) was 35–50% (SD: 19–28%). There were no significant differences in terms of achievement of ROSC and survival to hospital discharge (BF < 5 for both groups).

**Table 1:** Quality of chest compressions and ROSC in percent (%)

		ROSC		Bayes factor	Live discharge		Bayes factor
		Yes	No		Yes	No	
Depth	< 5 cm	24.50 (22.29)	28.87 (22.62)	0.421	35.16 (28.18)	33.19 (26.52)	0.217
	**5–6 cm**	**41.89** (20.61)	**41.94** (19.61)	0.139	**37.70** (19.66)	**37.25** (21.92)	0.204
	> 6 cm	33.61 (28.61)	29.19 (27.55)	0.286	27.14 (30.32)	29.55 (29.31)	0.220
	Mean	5.63 (0.83)	5.49 (0.77)	0.376	5.40 (0.89)	5.42 (0.94)	0.204
Rate	< 100/min	11.56 (18.70)	11.18 (16.64)	0.151	12.85 (22.27)	19.86 (24.66)	0.568
	**100–120/min**	**49.88** (25.06)	**48.30** (27.66)	0.147	**44.59** (29.14)	**44.62** (26.57)	0.203
	> 120/min	38.56 (27.91)	40.51 (30.30)	0.139	42.56 (32.93)	35.52 (28.34)	0.404
	Median/min	114.39 (12.26)	116.46 (11.04)	0.212	116.36 (13.24)	111.46 (14.12)	0.912

ROSC was achieved in 49.8% of patients (*n* = 543). In addition, 41.4% of patients (*n* = 451) were transported to a hospital, and 14.3% (*n* = 156) were transported with CPR in progress. Of the patients included in this study, 97.5% (*n* = 1063) received further treatment in a certified CAC. A total of 24.7% of patients (*n* = 269) survived the first 24 h, and 17.7% (*n* = 193) survived to hospital discharge. Their median Cerebral Performance Category (CPC) score was 2.

**Fig. 1:**
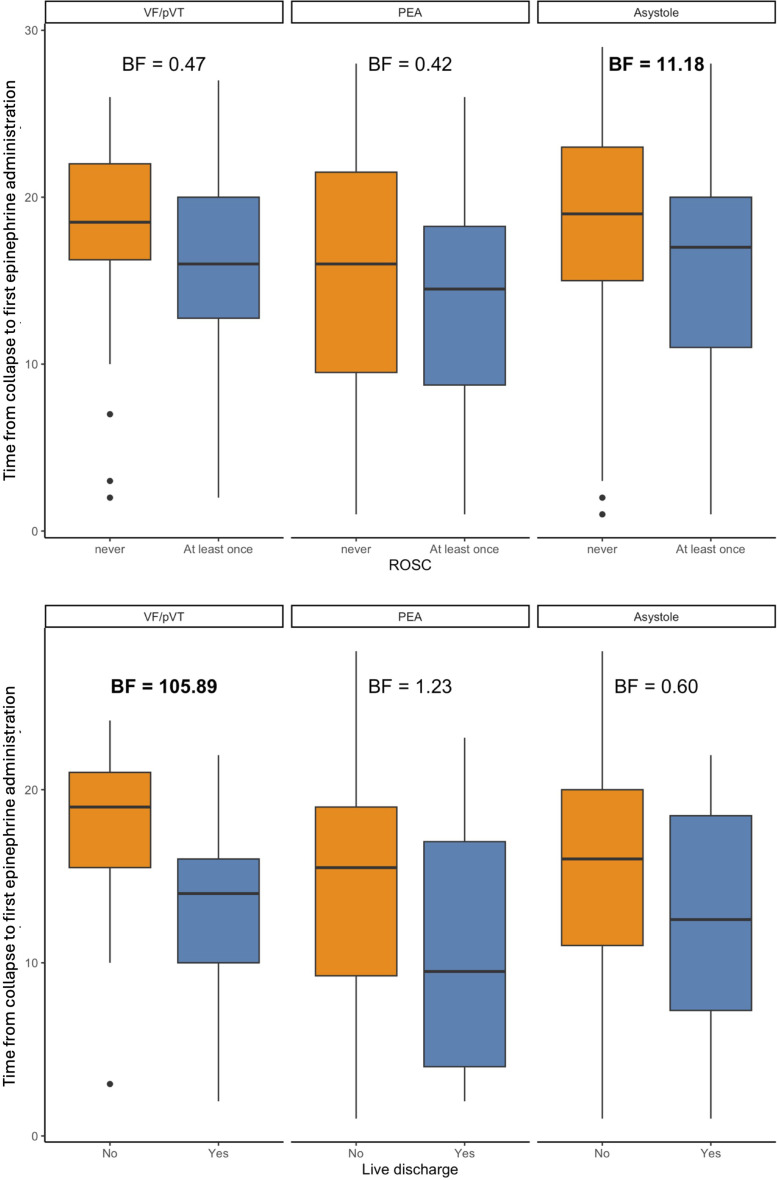
Time from collapse to epinephrine administration in minutes (min) and outcome

**Fig. 2:**
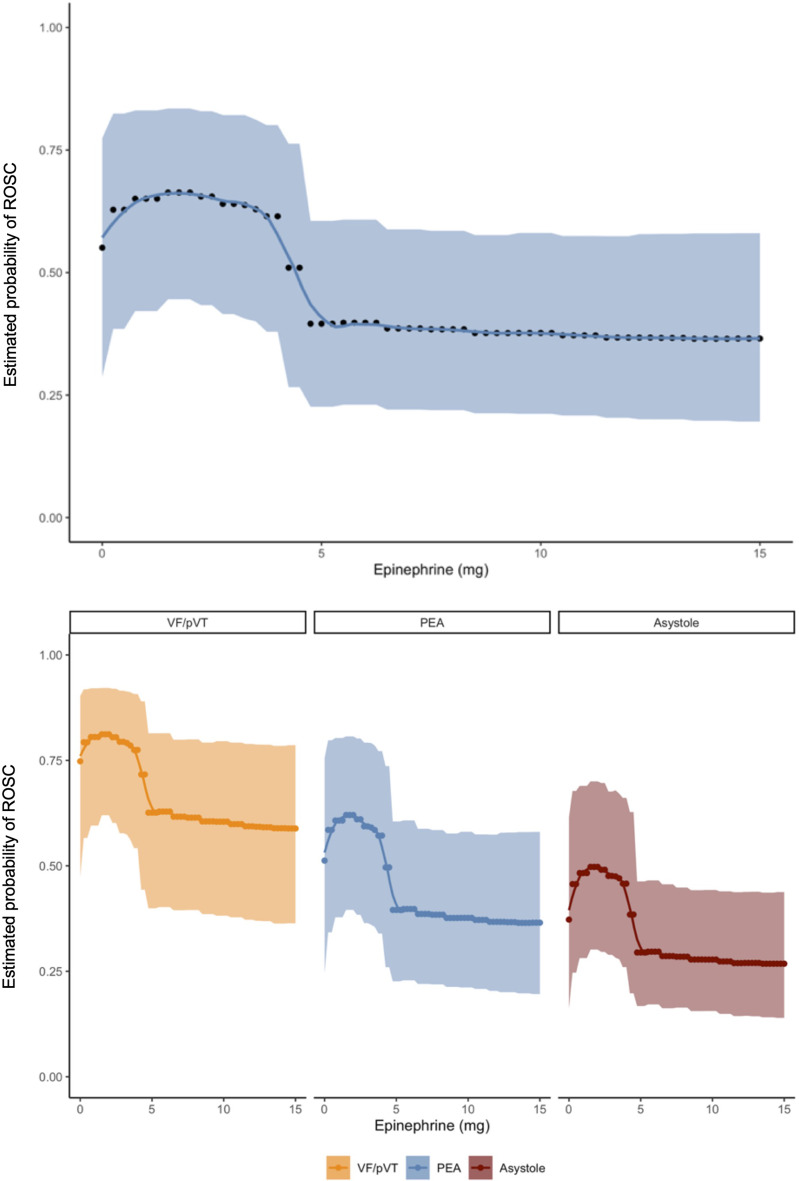
Probability of ROSC and total dose of epinephrine administered

**Fig. 3:**
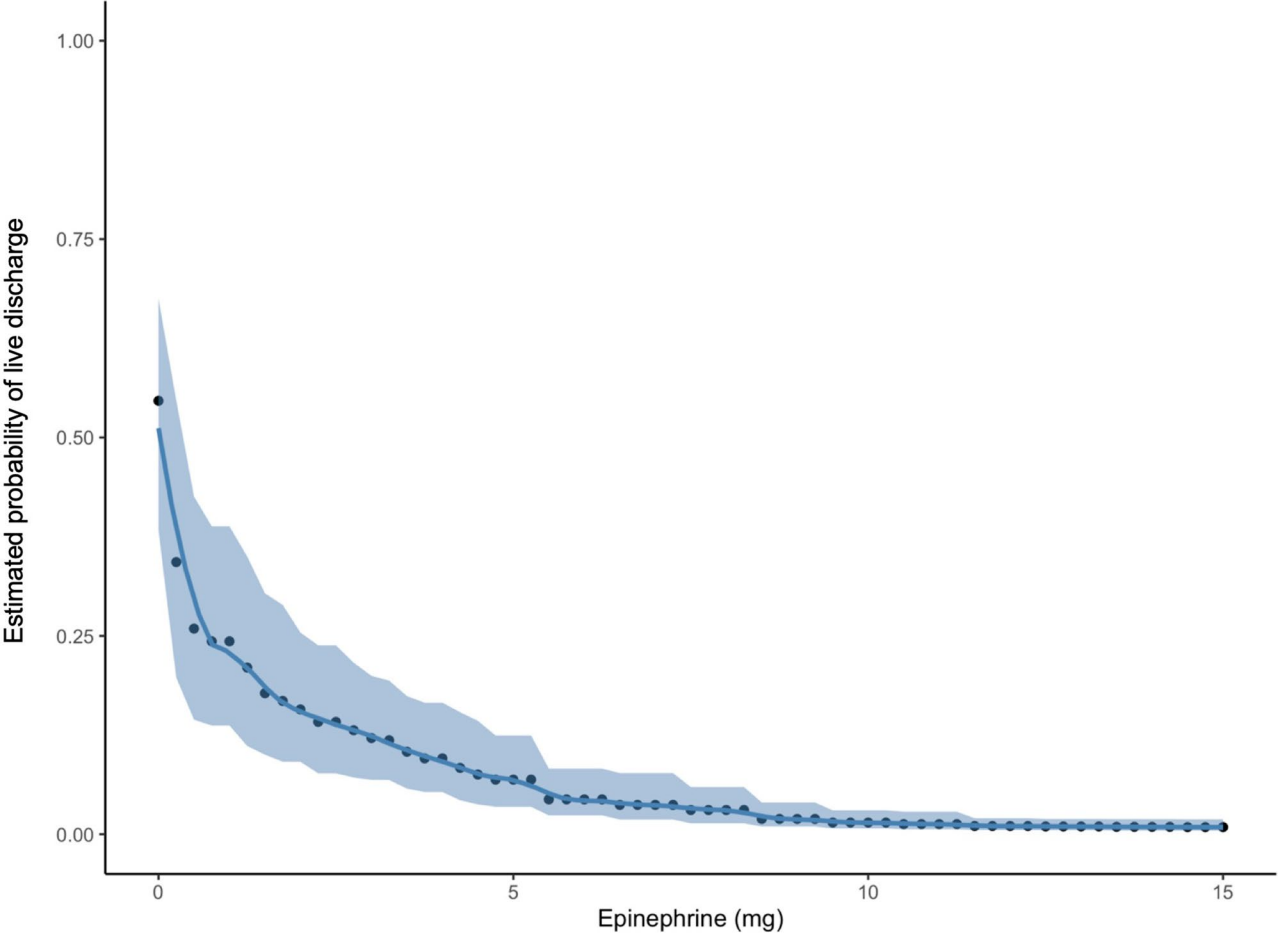
Probability of survival and total dose of epinephrine administered

**Fig. 4:**
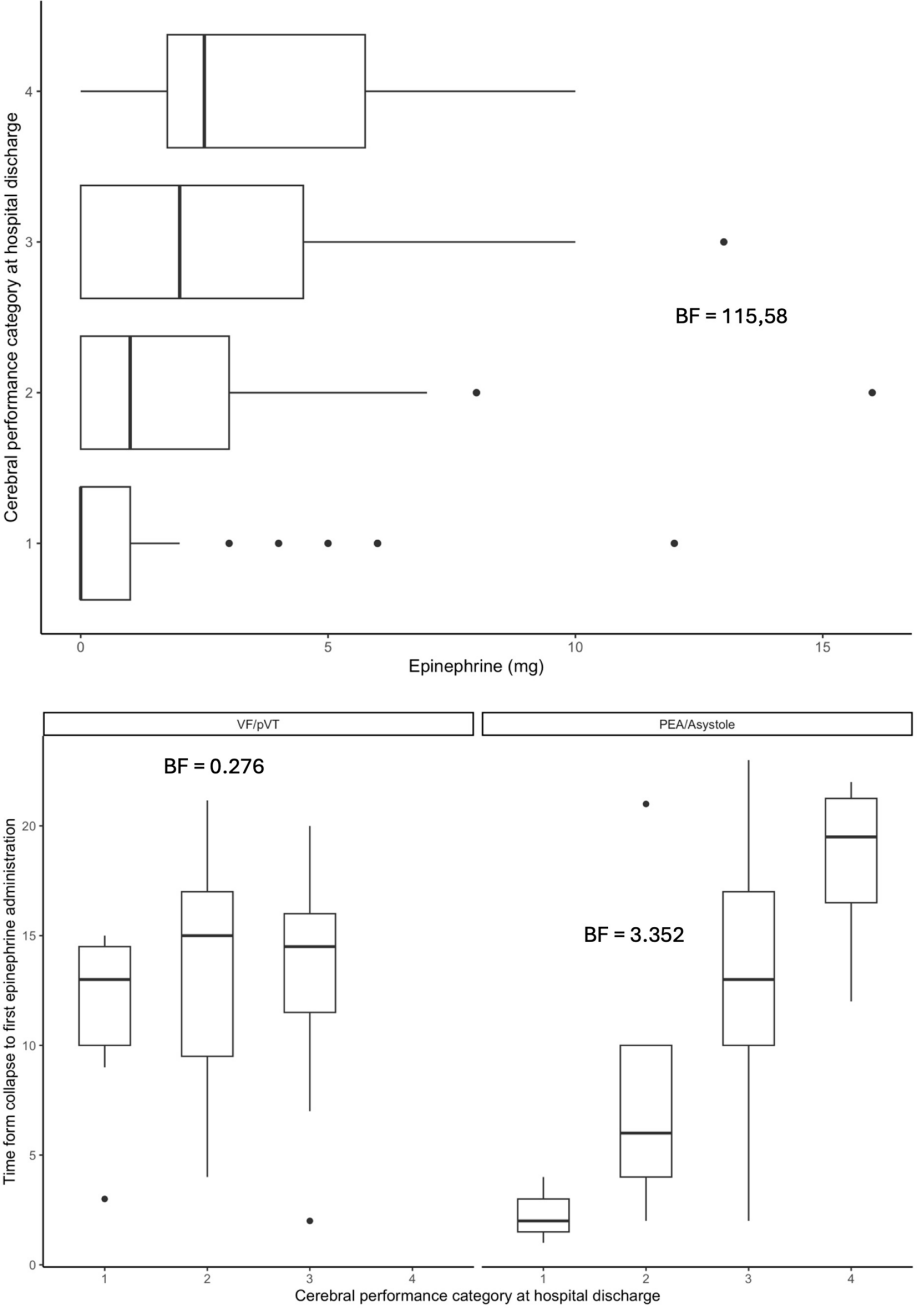
Neurological status at discharge and total dose of epinephrine administered or time from collapse to first epinephrine administration

Figure 1 shows that the earlier epinephrine was administered, the better the outcomes tended to be. Early administration of epinephrine after collapse was associated with significantly higher rates of ROSC in patients with asystole (BF = 11.18) and significantly higher rates of survival to hospital discharge in patients with an initially shockable rhythm (BF = 105.89). Moreover, significantly higher rates of ROSC were achieved after early epinephrine administration in patients with asystole who received IO access (BF = 15.10). It was not possible to perform a statistical analysis separately for epinephrine and amiodarone and to assess their specific influence on the higher rate of survival to hospital discharge which was observed in patients with shockable rhythms following early epinephrine administration. Epinephrine tended to have negative effects on survival, but the combination of both medications was generally administered in accordance with the European Resuscitation Council (ERC) Guideline recommendations [8]. Fig. 2 demonstrates an increased probability of ROSC at epinephrine doses ranging between 0 and 5 mg. The same model, however, did not show a similar effect of total epinephrine doses or the administration of epinephrine at a dose of 1 mg every 3 to 5 min on survival to hospital discharge for OHCA patients (Fig. 3). Likewise, IO access was not associated with this positive effect. Figure 4 suggests that neurological status at hospital discharge was less favourable with increasing total doses of epinephrine or with an increasing number of epinephrine doses during CPR in patients with OHCA. A trend towards better neurological outcomes following early administration of epinephrine was found especially in patients with non-shockable rhythms.

## Discussion

There is some controversy about the use of vasopressors in the management of prehospital and inhospital cardiac arrest. The study presented here investigates a group of OHCA patients who are comparable to those reported in international studies and who underwent CPR that was provided by a physician-staffed EMS. The physician-staffed EMS is a special feature of this study and can lead to improved mortality and morbidity, especially in emergencies, as the meta-analysis of Lavery et al. indicates [27]. Outcomes were as expected for the setting.

The results are further evidence of the benefits of early administration of epinephrine in patients with cardiac arrest. The majority of available studies report that early epinephrine administration is also associated with higher rates of ROSC [28–33]. It is interesting to note that the present study suggests that this positive effect appears to be independent of the initial electrocardiographic rhythm (Figs. 1 and 2) and is thus also seen in patients with shockable rhythms (VF/pVT). The present study does not provide conclusive evidence as to whether this effect in patients with shockable rhythms is attributable to either epinephrine or amiodarone, which are usually administered in combination. The vascular activity of epinephrine administered at a bolus of 1 mg may be the primary effect of epinephrine in patients with OHCA, and possible arrhythmogenic side effects may be of low relevance. Studies investigating the usefulness of vasopressin given as an alternative or an adjunct to epinephrine have reported even more heterogeneous results. Some studies demonstrated a benefit of vasopressin over epinephrine [34–38]. Other studies showed better results for the combined administration of both vasopressors [39–45]. A similar number of studies found no difference [46–51]. In addition, some recent studies identified benefits of the addition of steroids to epinephrine and/or vasopressin [52–55]. A substantial body of evidence exists on this subject, but recently, the use of vasopressin and prednisolone at least during intra-hospital resuscitation has shown only an increased rate of RSOC without significant benefit to survival or neurologic outcome for the patients [56–59]. In 2014, Donnino et al. conducted a post-hoc analysis and investigated effects of the timing of epinephrine administration during resuscitation [34]. The time of administration, however, appears to play a decisive role in the probability of survival and neurological outcome of patients with cardiac arrest. An analysis of data from the PARAMEDIC2 trial too confirmed the positive effects of early epinephrine administration on the achievement of ROSC [60]. 

Other routes of administration may be more rapid and may lead to better survival rates, as was demonstrated by Palatinus et al. in 2024. As a result of the shift of blood to the central circulation and the decreased perfusion of the skin and muscles, the delivery of epinephrine is difficult to assess after intramuscular administration but is associated with a persistent effect [61]. IO access too can be obtained more rapidly and thus allows medications to be delivered more rapidly. IO access is usually used only as a secondary route of application, if, for example, attempts at IV access have failed or a venous puncture site has not been found or because the IO route is associated with a longer absorption time. For this reason, this effect on patient outcome may be absent [62–64]. In the study presented here, there was no advantage of early epinephrine administration after collapse on survival or neurological outcome.

The finding that epinephrine administration is associated with a higher probability of ROSC may be a beneficial effect, but the most important result for patients is that they survive at least until discharge or longer. So far, however, only a few studies have reported evidence for this effect [65–67]. Important differences between the data presented in this study and the Prehospital Assessment of the Role of Epinephrine: Measuring the Effectiveness of Drug Administration in Cardiac Arrest (PARAMEDIC2) trial, which was conducted by Perkins et al. in 2018 and is currently the most important study on this subject, are the far earlier administration of epinephrine and our survival rate, which is comparable to internationally reported rates. At present, the underlying reasons (more personnel at the scene, better training/performance, better equipment, etc.) are unknown. In addition, factors that may influence outcome (e.g. the quality of chest compressions) can now be better assessed and endotracheal intubation, which is the gold standard in airway management, was performed in far more cases in the study presented here. Nevertheless, the key points of the two studies are comparable.

In 2014, Patanwala et al. conducted a meta-analysis in order to determine whether epinephrine afforded any survival benefit. They compared epinephrine to no epinephrine (or placebo) and did not find a survival benefit with the use of epinephrine during resuscitation [68]. Doses higher or lower than that recommended in pertinent guidelines (1 mg every 3 to 5 min) do not appear to provide any survival benefit [69–72]. Other studies reported that the quality of survival and the neurological status of patients who survived to discharge was poorer in patients who were treated with epinephrine, especially if they received higher cumulative doses [67, 73–76]. The study presented here also supports this finding. It does not allow definitive conclusions to be drawn on whether the key factor is the actual or total epinephrine dose or the duration of cardiac arrest in association with the more frequent use of the recommended boluses of epinephrine. The exact effect of epinephrine on cerebral perfusion must be investigated more thoroughly. The study, however, provides further prognostic evidence on when CPR is likely to be associated with a negative prognosis at least in patients with OHCA.

The results presented here support existing data, but the study has several limitations. Retrospective studies often do not accurately reflect real events and are subject to resuscitation time bias. This applies in particular to dynamic situations such as CPR in the prehospital setting. In many cases, the time when a specific intervention was carried out at the scene was probably estimated and documented by an EMS team after the mission. In addition, it is impossible to accurately assess the main effect (on survival) of an intervention that in general is beneficial for patients or in the treatment of patients when a patient’s final outcome is strongly influenced by adverse factors (e.g. no-flow time, underlying conditions such as ruptured aortic dissection). Nevertheless, the data collected from a relatively large sample of OHCA patients who were treated by a physician-based EMS support a trend that should be further investigated with a view to assessing in greater detail the use of vasopressors during resuscitation.

A special feature of this study was the inclusion of the quality of basic life support first time by measuring the frequency, depth and relief rate of chest compressions using a feedback system placed on the chest of the patients. The effect of all study trials can only be validly assessed if the effective basic measures known to us today are correctly performed. The findings of this study do not provide evidence of a benefit of high-quality chest compressions. The same applies to other studies that measured coronary perfusion pressure and did not report an effect on the probability of discharge alive or on neurological outcome [77]. The restoration of minimal circulation in the resuscitation setting can maybe result in a early reperfusion injury, which can lead to the accumulation of acid metabolites in a situation in which the underlying problem is not or cannot be treated. Immunological factors and oxygen radicals disrupt cell function or even lead to cell death and these mechanisms occur after global ischemia during cardiac arrest in almost all cells such as the brain, heart, kidneys and vascular system [78, 79]. This may have more adverse than beneficial effects. Against this background, the importance of chest compressions during CPR should be discussed from an entirely new perspective. In 2023, for example, Trummer et al., who conducted a study to evaluate the impact of head elevation during reperfusion in a swine model, achieved good neurological recovery after twenty minutes of cardiac arrest without chest compressions [80]. However, this hypothesis has not been sufficiently developed yet and needs to be investigated further. CPR should be performed in accordance with the currently guidelines and medication should be administered in that way until then. So as long as the administration of vasopressors is recommended during CPR, further studies should be conducted to address also this issue about the best way of implementation.

## Conclusions

The results reported here support the benefits of early epinephrine administration for OHCA patients in a physician-staffed emergency medical service system. It appears, however, that beneficial effects only occur if a certain total dose of epinephrine or number of epinephrine boluses or, in other words, a certain duration of CPR or OHCA are not exceeded. Furthermore, epinephrine administration primarily leads to an increase in ROSC rates, which, of course, is the first step to survival. In conclusion, the results of this study add to existing data, and randomised controlled trials are warranted to provide definitive evidence. The exact biological effect of vasopressors on cerebral perfusion as an important outcome of CPR has not yet been fully clarified.

## Supplementary Information

Below is the link to the electronic supplementary material.

Supplementary Material 1

Supplementary Material 2

Supplementary Material 3

## Data Availability

The datasets used and/or analysed during the current study are available from the corresponding author on reasonable request.

## Abbreviations

BART: Bayesian additive decision trees
CAC: Cardiac arrest centre
CPC: Cerebral Performance Category
CPR: Cardiopulmonary resuscitation
ECG: Electrocardiogram
EMS: Emergency medical services
ERC: European Resuscitation Council
IO: Intraosseous
IV: Intravenous
mg: Milligram
min: Minutes
OHCA: Out-of-hospital cardiac arrest
PEA: Pulseless electrical activity
PES: Pre-emergency status
pVT: Pulseless ventricular tachycardia
ROSC: Return of spontaneous circulation
SD: Standard deviation
VF: Ventricular fibrillation
