# Supplementary material for: The use of epinephrine in out-of-hospital cardiac arrest: A retrospective study of the effects of administration timing and cumulative doses on outcome in a physician-staffed emergency medical service system
Source: BMC Emerg Med. 2025 Sep 22;25:182. doi: 10.1186/s12873-025-01351-4 (PMC12455778; doi:10.1186/s12873-025-01351-4)
Supplement: Supplementary file 3 — Supplementary Material 3 [file 12873_2025_1351_MOESM3_ESM.pptx]

## Slide 1
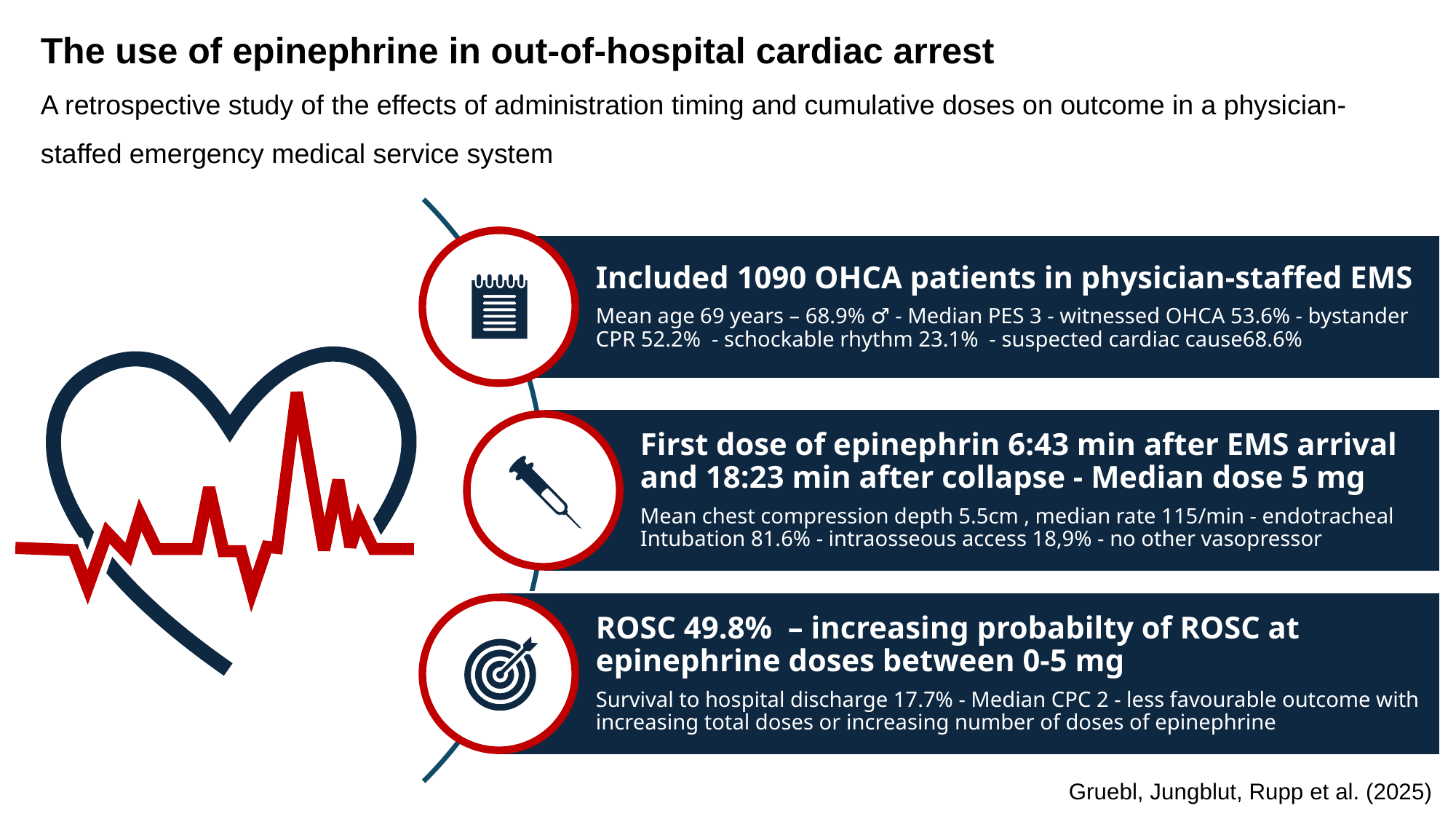

# The use of epinephrine in out-of-hospital cardiac arrest A retrospective study of the effects of administration timing and cumulative doses on outcome in a physician-staffed emergency medical service system
Gruebl, Jungblut, Rupp et al. (2025)
